# Supplementary material for: Chromothripsis during telomere crisis is independent of NHEJ, and consistent with a replicative origin
Source: Genome Res. 2019 May;29(5):737–49. doi: 10.1101/gr.240705.118 (PMC6499312; doi:10.1101/gr.240705.118)
Supplement: Supplemental Material [file supp_gr.240705.118_Supplemental_file_1.zip › contigs/annotated_contigs/DB57/contig.2.DB57_length_329_mean_cov_8.30699088146.docx]

**DB57_length_329_mean_cov_8.30699088146**

```
TGTGTTGGGCTCCACGTTTCTGTC|TTCTGTCTTCTGTCTTCTATCACTGAGGTTTTATTCCTATCCTTGTTGTTTTCTATAGTTTCTT
                         >chr9:68417363-68417602 - E=1e-117
CTATTATGTCTTTTTCTGTCTTGTGATTTGAAAACTATATGACACATTGTAATGTTTTAATTTTTCTAATTTTTTGCTTTTAATTTTAA

CTAATTTTATTTTTTAGAGAAGTGCAGGTTCACAGCTAAACGGAGCAGAGAGTACAGACTTCTCATATGTCCCTTTCCCCACA|CAC|T
                                                                                    >ch
CAAAGCTTACAGTGCACATTCACGTTTACTCTTCACGTTGTACGTTCTGAGTCTTGACAAAAGTAr9:68417218-68417287 - E=4e-30
```
